# Parathyroid Hormone Levels in the Prediction of Ischemic Stroke Risk

**DOI:** 10.1155/2017/4343171

**Published:** 2017-01-02

**Authors:** Güner Çelik, Ali Doğan, Şefik Dener, Şerefnur Öztürk, Sevsen Kulaksızoğlu, Hakan Ekmekçi

**Affiliations:** ^1^Beyhekim State Hospital, Department of Neurology, Konya, Turkey; ^2^Bandırma State Hospital, Department of Neurology, Balıkesir, Turkey; ^3^Department of Neurology, Başkent University, Konya Training and Research Hospital, Konya, Turkey; ^4^Department of Neurology, Selçuk Medical Faculty, Selçuk University, Konya, Turkey; ^5^Department of Biochemistry, Başkent University, Konya Training and Research Hospital, Konya, Turkey

## Abstract

*Objective*. It was examined whether PTH and 25-dihydroxyvitamin D (25(OH)D) levels, together or separately, are indicators of the risk of stroke.* Materials and Methods*. This prospective study was performed at two centers. In the study, 100 patients diagnosed with acute ischemic stroke and 100 control individuals in the same age range were examined. In addition to neurological examination, cranial imaging, extensive routine blood chemistry, PTH, and 25(OH)D levels were evaluated in all cases. Stroke risk factors were determined. Logistic regression was used for statistical analysis.* Results*. A total of 60 patients and 79 control individuals were included in the study. Different estimation models were designed in order to examine the relationship between PTH and 25(OH)D levels with stroke. According to modeling results, it was determined that the most effective predictor for risk of stroke was 25(OH)D levels, followed by hypertension and PTH levels, respectively.* Conclusion*. PTH and 25(OH)D levels together can make important contributions to determination of stroke risk, and further investigations are needed to understand this relationship more fully.

## 1. Introduction

The key role of parathyroid hormone (PTH) in bone health and homeostasis is well known. However, recent studies have indicated that PTH has various effects on other organs and tissues like 25(OH)D. PTH shows the effect of PTH receptors in tissues through which receptors are expressed in smooth muscle cells on the vascular wall, endothelium, and myocardium [1]. Its level is frequently increased by 25(OH)D (25-dihydroxyvitamin D) deficiency or, to a lesser extent, chronic renal failure. However, some studies have demonstrated that elevated PTH levels are common even in healthy people with neither 25(OH)D deficiency nor chronic renal failure [2]. Elevated PTH levels raise blood pressure and cardiac contractility, resulting in fibrosis, apoptosis, and hypertrophy in cardiomyocytes of the left ventricle and vascular smooth muscle cells [3]. Several recent studies have demonstrated that it is associated with various cardiovascular conditions such as endothelial dysfunction, vascular stiffness, and calcification [4], increased aortic pulse pressure [5], reduced great artery elasticity [6], coronary microvascular dysfunction, and hypertension [7]. Furthermore, it has also been shown that increased PTH levels may affect the cardiovascular system by stimulating cytokine release from lymphocytes and vascular smooth muscle cells [1, 8]. It has been argued that PTH confers a risk for cardiovascular disease even at normal or slightly elevated levels and in the absence of mineral metabolism disorders. Since all these data suggest that increased PTH levels may increase risk for cerebrovascular disease, the aim of this study was to investigate the correlation between PTH levels and stroke and to study 25(OH)D because of its close correlation with PTH.

## 2. Materials and Methods

This prospective study was conducted in two separate centers between 2010 and 2014. The study group included 100 patients admitted to emergency department or outpatient clinics for symptoms of acute stroke and were diagnosed with acute ischemic stroke using cranial imaging methods. The control group consisted of 100 age-matched subjects with completely normal MRI cranial examination results, no history of stroke or transient ischemic attack, and no signs or symptoms of cerebrovascular disease. In both groups, subjects using supplemental calcium or vitamin D were excluded, as well as those with chronic renal failure, chronic liver disease, or osteoporosis.

In addition to obtaining a detailed medical history and performing a thorough neurological examination, either cranial magnetic resonance imaging (MRI) or computerized tomography (CT) was obtained for differential diagnosis at admission in all subjects. Age, gender, arterial blood pressure, and electrocardiography were recorded for all subjects in both groups. Additionally, comprehensive blood biochemistry and levels of 25(OH)D and PTH were measured in all subjects. Risk factors for stroke (hypertension (HT), diabetes mellitus (DM), atrial fibrillation (AF), coronary artery disease (CAD), history of myocardial infarction (MI), tobacco or alcohol use, and obesity) were recorded. Blood samples were obtained by venipuncture after an overnight fast and placed into tubes that were protected from sunlight. Sera were separated and stored at −80°C within 30 min of collection. Serum PTH levels were determined by a chemiluminescent microparticle immunoassay (CMIA) method in an Abbott Architect i2000 analyzer using the Abbott Architect Intact PTH assay kit; the reference range was set at 10–65 pg/mL. The same method was also used to determine 25(OH)D levels. The reference range for 25(OH)D was set at 10–55 *μ*g/L.

This research project was approved by the local ethics committee.

### 2.1. Statistical Analysis

Since the data regarding ages and PTH levels was normally distributed, a two-sample independent *t*-test was used to compare the two groups (patient and control groups). 25(OH)D levels were not normally distributed, and thus the nonparametric Mann–Whitney *U* test was used in order to compare the two groups. Gender distribution between the two groups was evaluated using the chi-square test. Logistic regression analysis was used in order to properly estimate the stroke patients and healthy individuals (*n* = 139). Three appropriate logistic regression models were designed using efficient and significant predictors. In the first model, 25(OH)D and PTH levels were used for estimation of stroke risk. In the second model cardiac risk factors (AF, CAD, and MI) were used in addition to 25(OH)D and PTH levels for stroke risk estimation. In the third model, 25(OH)D and PTH levels as well as all cardiovascular risk factors were evaluated. For these models, the formula of hazard ratio (HR) was used. For example, HR for Model 1.3 is

(1) $$\begin{array}{c} \pi \left(\;{OH}_{25};\text{PTH}\;\right)=1-\frac{e^{-0\text{.}757+0\text{.}113\left({OH}_{25}\right)-0\text{.}013\left(\text{PTH}\right)}}{1+e^{-0\text{.}757+0\text{.}113\left({OH}_{25}\right)-0\text{.}013\left(\text{PTH}\right)}}. \end{array}$$

If 25(OH)D and PTH levels are 11.9 and 164.7 in the patient, respectively, ischemic stroke risk is estimated as 83.4%.

(2) π11.90;164.71−e−0.757+0.11311.9−0.013164.71+e−0.757+0.11311.9−0.013164.7=1−0.1659=83.4%.

If 25(OH)D and PTH levels are 19.20 and 60.3 in the patient, respectively, ischemic stroke risk is estimated as 35.3%.

(3) π19.20;60.301−e−0.757+0.11319.20−0.01360.301+e−0.757+0.11319.20−0.01360.30=1−0.6463=35.3%.

The study power for 25(OH)D measurements was 92%, using a standard deviation of 8.05 and difference value of 5, with 60 patients in each group. For PTH measurements, the study power was 79% using a standard deviation of 38.91, difference value of 20, and 59 patients in each group. Minitab Release 14.0 and SPSS 15.0 for Windows (SPSS, Inc., Chicago, IL) statistical programs were used for statistical analyses.

## 3. Results

This study included a total of 200 subjects: 100 patients with acute stroke and 100 control subjects. Subjects with problematic blood sampling, storage, or analysis for PTH and 25(OH)D levels were excluded from the study. Subjects with extreme measurement values who created heterogeneity in the distribution of both groups and those who were difficult to age-match between the groups (the extremely old or young) were excluded. Obese subjects and those with habits of tobacco or alcohol use were also excluded due to the small number and unequal distribution of these individuals across the groups. After the completion of the above exclusion procedures, data from a total of 60 patients and 79 control subjects remained in the data set used for 25(OH)D analysis. PTH analysis was performed after excluding 1 more subject with PTH levels of approximately 500 pg/mL.

No significant difference was found between the mean age and gender distribution of both groups (Tables 1 and 2).

As shown in Table 3, the mean 25(OH)D level was significantly lower in the patient group (15.7 ± 4.27) compared to the control group (20.1 ± 8.05) (*Z* = 3.147, *p* = 0.002). In contrast to the vitamin D level, the PTH level was significantly higher in the patient group (82.83 ± 38.91) compared to the control group (64.74 ± 28.80) (*t* = −2.998, *p* = 0.002). These two comparisons were strongly significant.

**Figure 1:**
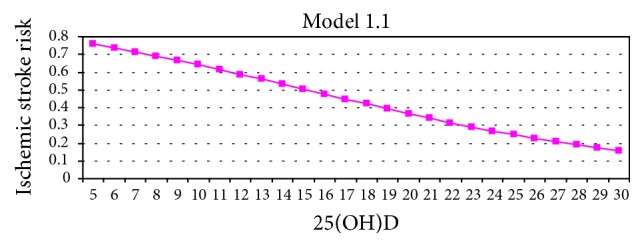


**Figure 2:**
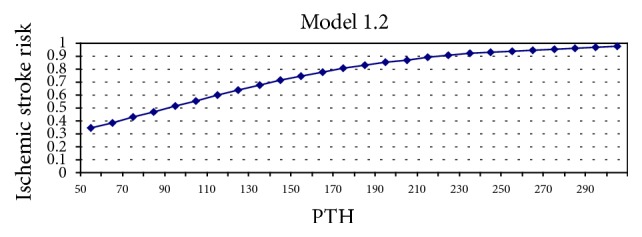


Different prediction models were used to examine the association between stroke and PTH, 25(0H)D levels. First, it was determined whether PTH and 25(OH)D could be used as markers for predicting stroke risk (Table 4). The abilities of 25(OH)D and PTH levels, both alone and in conjunction, to accurately predict stroke patients and healthy subjects were tested, as outlined by Model 1. According to this model, 25(OH)D levels had an accurate prediction rate of 48.3% for stroke patients and 70.9% for healthy subjects; it has an overall accurate prediction rate of 61.2% (wald = 12.215, *p* = 0.000). When the PTH level was used as the prediction marker, the accurate prediction rate was 35.6% for stroke patients, 81.8% for the controls, and 61.8% as an overall accurate prediction rate (wald = 8.129, *p* = 0.004). Models 1.1 and 1.2 are presented in Figures 1 and 2 graphically. When PTH and 25(OH)D were analyzed together (Model 1.3), the accurate prediction rate increased to 57.6% and the overall accurate prediction rate increased to 64%. The 20% increase in accurate prediction rate indicated that both factors were more effective for accurate prediction when used in conjunction (wald = 4.822, *p* = 0.028). Ten percent of 30 patients incorrectly diagnosed by 25(OH)D were accurately categorized by PTH (Model 1.4) in a statistically significant manner (wald = 3.911, *p* = 0.048).

25(OH)D and PTH, both alone and in conjunction, were also used as prediction tools in the presence of cardiac risk factors in Model 2 (Table 5). In this model, 25(OH)D was used in conjunction with risk factors such as CAD, MI, and AF (Model 2.1). The accurate prediction rate of the model was 53.3% (*p* = 0.001) for the stroke patients and 76.9% (*p* = NS) for the healthy subjects. When used in conjunction with cardiac risk factors, the accurate prediction rate of PTH was 45.8% (*p* = 0.41) (Model 2.2). This result was also statistically significant, although it was weaker than that of 25(OH)D. The accurate prediction rate for the healthy subjects was 90.8% (*p* = NS). When both 25(OH)D and PTH were used in conjunction with other cardiac risk factors (Model 2.3), the accurate prediction rate was the same as that obtained without taking cardiac risk factors into account (57.6%) (*p* = 0.99). Among all risk factors considered, CAD significantly contributed to accurate prediction. However, this effect was significantly weaker than those of 25(OH)D and PTH. Of 28 patients not accurately predicted by an analysis using cardiac risk factors and 25(OH)D together, only 7.1% were accurately predicted by PTH and IHD, and this difference did not reach statistical significance.

Model 3 used 25(OH)D and PTH in conjunction with other cardiovascular risk factors (HT, DM, and lipid levels) in addition to the cardiac risk factors studied in Model 2.  Table 6 shows the effect and statistical significance of the predictors used in this analysis. The model in which 25(OH)D was used with all of these risk factors (Model 3.1) had an accurate prediction rate of 68.3% for the stroke patients. Compared to all other factors, 25(OH)D had the greatest effect on the accurate prediction rate (*p* = 0.002). The effect of HT was smaller, but statistically more significant than that of 25(OH)D (*p* = 0.010). Other risk factors had no effect on the accurate prediction rate. When PTH was used with all cardiovascular risk factors (Model 3.2) the accurate prediction rate was 59.3%. In this analysis, the effect of PTH on the result was statistically significant (*p* = 0.019), but less than that of HT. The most powerful effect in the analysis was that of HT (*p* = 0.002). Other risk factors had no effect on the result. When both 25(OH)D and PTH were used together with all other risk factors (Model 3.3), the accurate prediction rate was found to be 69.5%. In this analysis it was observed that both 25(OH)D and HT were two important indicators for predicting strokes, with the effect of 25(OH)D being more powerful than that of HT. Furthermore, effect of PTH was minimal and not statistically significant in this analysis. Model 3.4, in which PTH and other risk factors were used, was not successful for nineteen patients who were incorrectly diagnosed using 25(OH)D levels. In conclusion, a combined review of all analyses revealed that 25(OH)D was the most important factor for stroke prediction, followed by HT and PTH, in descending order. The effect of CAD was minimal and not statistically significant.

## 4. Discussion

In examining the association between serum PTH levels and cerebrovascular disease, as well as the contribution of 25(OH)D, this study made some important conclusions. First, serum PTH levels were significantly higher and 25(OH)D levels were significantly lower in the patient group compared to the control group. Numerous studies have stressed the association between serum 25(OH)D levels, cardiovascular disorders [9, 10], and mortality [11]. It has also been shown that 25(OH)D levels are lower in patients with stroke [12, 13]. Moreover, 25(OH)D deficiency increases risk of stroke and even affects stroke prognosis [14], causing more stroke-related fatal outcomes [15]. In contrast, the number of studies examining the association between increased PTH levels and cardiovascular diseases [6, 16], mortality [11, 17], and particularly, stroke [18, 19] is quite limited. Of these studies, one conducted by Sato et al. detected a decrease in serum 25(OH)D levels and BMD values and an increase in serum ionized calcium and PTH levels in female subjects with ischemic stroke. In the patient group the incidences of hypertension and coronary artery disease were higher than in the control group, as was the prevalence of lacunar infarcts; that group also entered menopause earlier [20]. A study examined the association between stroke and PTH in patients with hypercalcemia, and elevated PTH levels were associated with stroke, the incidence of which was 7.1% in patients with primary hyperparathyroidism [19].

The second finding of our study was that both PTH and 25(OH)D levels were two important markers that could be used to predict the future risk of stroke. Of these two predictors, 25(OH)D was a fairly powerful marker, while PTH was weaker, although still statistically. When both predictors were used together, the accurate prediction rate for stroke was further increased. This demonstrated that PTH is an important marker for defining stroke risk, but its predictive power is enhanced when used in conjunction with 25(OH)D. This suggests that combined use of these two markers would be a more powerful marker in risk assessment.

There exist some studies relating low 25(OH)D levels to increased risk for all cardiovascular disorders, including stroke [10, 21, 22], MI, and carotid atherosclerosis [14, 23]. However, studies have predominantly reported that increased PTH caused vascular abnormalities rather than stroke and mentioned it as a risk factor for cardiovascular disorders.

In two Swedish population studies of more than 1000 patients over 70 years of age, Hagström et al. reported that PTH was a powerful predictor for both clinical and subclinical atherosclerosis. In this study, plasma PTH levels above 50 pg/mL conferred a 20% risk for cardiovascular mortality [24]. In addition, several studies have supported the notion that increased PTH levels cause atherosclerosis or vessel wall dysfunction [16, 25, 26].

Anderson et al. found that PTH levels were higher in subjects with increased prevalence of cardiovascular risk factors (HT, DM). They also observed that PTH levels were elevated before other risk factors were apparent, which suggests that PTH may contribute to the development of such risk factors [2]. Another study found that increased serum PTH levels were correlated to the number of stenotic arteries, HT, and low ejection fraction.

In a population-based study by Wannamethee et al. examining the association between heart failure (HF) and PTH, increased PTH levels were correlated with HF risk, although such a risk was not related to mineral metabolism and 25(OH)D. This was explained by the hypothesis that, in the absence of chronic renal failure, PTH exerts its cardiac actions via PTH receptors found in myocardium [1]. In a study by Bansal et al. increased serum PTH levels were correlated with increased risk of HF and left ventricular mass, although such an association was absent for 25(OH)D [27].

Multiple studies have shown that PTH was predictive for vascular disease and death associated with disorders of mineral metabolism including primary and secondary hyperparathyroidism and CRF [18, 28, 29]. Hagström et al. explained the association between PTH and atherogenesis in the following way: vascular calcification and remodeling result from direct PTH receptor interaction on the vessel wall, indirect inflammation, and vascular dysfunction. In addition, increased PTH levels are associated with inflammation markers, which are now considered cardiovascular risk factors [29]. The observation of a decreased incidence of CV disorders after the reduction of PTH levels by parathyroidectomy, renal transplantation, or calcimimetic agents supports the causal role of PTH in the development of CV disorders [29].

The third result of our study was that PTH, 25(OH)D, and HT were the most powerful markers for the prediction of stroke risk, even when all other cardiovascular risk factors were included in the analysis. When these three markers were compared with one another, 25(OH)D was the most powerful predictor, followed by HT and PTH in descending order. The weakest predictor was CAD, which has no significant predicting ability in the presence of the above three markers. Schierbeck et al. found that both PTH and vitamin D were independently associated with both cardiovascular and all-cause mortality [30]. These findings suggest that although both vitamin D and PTH appear to be separate risk factors for both stroke and all cardiovascular disorders combined, examining the two predictors in conjunction provides more accurate risk assessment for cardiovascular disorders. Our study limitation was the exclusion of an older patient group from the analysis to ensure equality in patient and control groups, which led to a relatively younger study population and, therefore, a potential bias.

In conclusion, PTH levels were increased, while 25(OH)D levels were decreased in patients with stroke. Both PTH and vitamin D appear to be separate risk factors for stroke. 25(OH)D was the most powerful marker for the predicting the stroke risk, followed by HT and PTH, in descending order. In addition to 25(OH)D, PTH serum levels should be considered, and both predictors should be assessed in conjunction for more accurate determination of stroke risk. Additional studies are needed to investigate the effect of PTH on stroke risk, the interaction of both predictors, and possible conditions that may develop as a result of dysregulated vitamin D and PTH synthesis.

## Figures and Tables

**Table 1:** Age distribution in the patient and control groups.

	Group	*n*	Mean	Std. deviation	Std. error mean	Test	*p*
Age	Patient	60	61.2833	6.55897	.84676	*t* = −0.759	0.449
	Control	79	62.2152	7.59668	.85469		

**Table 2:** Gender distribution in the patient and control groups.

		Gender		Total
		Female	Male	
Patient	Count	25	35	60
	% within group	41.7%	58.3%	100.0%
Control	Count	32	47	79
	% within group	40.5%	59.5%	100.0%

Total	Count	57	82	139
	% within group	41.0%	59.0%	100.0%

**Table 3:** Comparison of 25(OH)D and PTH levels between the patients and controls.

	Group	*N*	Mean	Std. deviation	Test	Sig
25(OH)D	Patient	60	15.7117	4.27599	*Z* = −3.147	0.002
	Control	79	20.1608	8.05743		
PTH	Patient	59	82.8373	38.91614	*t* = −2.998	0.002
	Control	77	64.7416	28.80296		

**Table 4:** Rates of accurate prediction of patients and disease-free subjects by 25(OH)D and PTH level (Model 1).

	*B*	SE	Wald	df	Sig.	exp⁡(*B*)	Accurate prediction rate of the patient group	Accurate prediction rate of the control group	Overall accurate prediction rate
Model 1.1									
25(OH)D	.113	.032	12.215	1	.000	1.119	48.3	70.9	61.2
Constant	−1.713	.577	8.814	1	.003	.180			
Model 1.2									
PTH	−.017	.006	8.129	1	.004	.983	35.6	81.8	61.8
Constant	1.477	.457	10.429	1	.001	4.379			
Model 1.3									
25(OH)D	.113	.034	10.903	1	.001	1.119	57.6	68.8	64.0
PTH	−.013	.006	4.822	1	.028	.987			
Constant	−.757	.790	.918	1	.338	.469			
Model 1.4 (the classification of 30 patients unclassified by Model 1.1)									
PTH	−.012	.006	3.911	1	.048	.988	10.0	98.7	73.8
Constant	1.810	.499	13.149	1	.000	6.111			

**Table 5:** Rates of accurate prediction of patients and disease-free subjects by 25(OH)D and PTH used in conjunction with cardiac risk factors (Model 2).

	*B*	SE	Wald	df	Sig.	exp⁡(*B*)	Accurate prediction rate of the patient group	Accurate prediction rate of the control group	Overall accurate prediction rate
Model 2.1									
25(OH)D	.130	.039	11.214	1	.001	1.139	53.3	76.9	66.7
CAD(1)	1.169	.567	4.245	1	.039	3.217			
MI(1)	21.211	12641.233	.000	1	.999	1628236695.777			
AF(1)	21.822	12669.616	.000	1	.999	3001279038.402			
Constant	−45.710	17897.485	.000	1	.998	.000			
Model 2.2									
PTH	−.013	.006	4.162	1	.041	.987	45.8	90.8	71.1
IHD(1)	1.319	.561	5.527	1	.019	3.738			
MI(1)	20.396	13141.236	.000	1	.999	721159600.690			
AF(1)	21.526	13937.969	.000	1	.999	2232101328.556			
Constant	−41.590	19156.176	.000	1	.998	.000			
Model 2.3									
25(OH)D	.132	.041	10.554	1	.001	1.141	57.6	81.6	71.1
PTH	−.009	.007	1.773	1	.183	.991			
CAD(1)	1.134	.582	3.795	1	.051	3.108			
MI(1)	20.780	12788.170	.000	1	.999	1058694576.339			
AF(1)	21.873	13324.999	.000	1	.999	3156046476.935			
Constant	−44.722	18468.707	.000	1	.998	.000			
Model 2.4 (the classification of 28 patients unclassified by Model 2.1)									
PTH	−.012	.007	2.694	1	.101	.988	7.1	98.7	74.3
IHD(1)	.361	.741	.237	1	.626	1.435			
Constant	1.513	.804	3.538	1	.060	4.540			

**Table 6:** Rates of accurate prediction of patients and disease-free subjects by 25(OH)D and PTH used in conjunction with all cardiac risk factors (Model 3).

	*B*	SE	Wald	df	Sig.	exp⁡(*B*)	Accurate prediction rate of the patient group	Accurate prediction rate of the control group	Overall accurate prediction rate
Model 3.1									
D_VIT_OH_25	.129	.042	9.325	1	.002	1.137	68.3	80.8	75.4
CAD(1)	.472	.642	.539	1	.463	1.603			
MI(1)	21.250	12597.619	.000	1	.999	1692925884.235			
AF(1)	21.894	12648.666	.000	1	.999	3224649359.264			
HT(1)	1.251	.484	6.691	1	.010	3.495			
DM(1)	−.255	.530	.232	1	.630	.775			
TRG	−.005	.004	2.421	1	.120	.995			
HDL	−.011	.025	.188	1	.665	.989			
LDL	.006	.007	.909	1	.340	1.006			
Constant	−44.958	17851.854	.000	1	.998	.000			
Model 3.2									
PTH	−.017	.007	5.467	1	.019	.983	59.3	82.9	72.6
CAD(1)	.649	.623	1.083	1	.298	1.913			
MI(1)	20.437	12784.724	.000	1	.999	751248842.438			
AF(1)	21.428	13795.882	.000	1	.999	2023693999.998			
HT(1)	1.468	.470	9.770	1	.002	4.341			
DM(1)	.294	.529	.309	1	.579	1.342			
TRG	−.003	.003	.752	1	.386	.997			
HDL	.017	.025	.453	1	.501	1.017			
LDL	.004	.007	.391	1	.532	1.004			
Constant	−42.241	18808.915	.000	1	.998	.000			
Model 3.3									
D_VIT_OH_25	.126	.044	8.225	1	.004	1.135	69.5	80.3	75.6
PTH	−.012	.008	2.377	1	.123	.988			
CAD(1)	.467	.659	.503	1	.478	1.595			
MI(1)	20.884	12515.881	.000	1	.999	1174192607.473			
AF(1)	21.839	13255.218	.000	1	.999	3050522065.125			
HT(1)	1.403	.501	7.848	1	.005	4.067			
DM(1)	.155	.561	.077	1	.782	1.168			
TRG	−.003	.004	.924	1	.337	.997			
HDL	.000	.027	.000	1	.995	1.000			
LDL	.006	.007	.696	1	.404	1.006			
Constant	−44.764	18230.411	.000	1	.998	.000			
Model 3.4 applied to 19 patients remained from Model 3.1									
PTH	−.011	.009	1.648	1	.199	.989	0.0	100.0	80.2
CAD(1)	.625	.856	.533	1	.465	1.868			
HT(1)	.243	.556	.190	1	.663	1.275			
DM(1)	−.640	.762	.704	1	.401	.527			
TRG	−.003	.004	.410	1	.522	.997			
HDL	−.006	.030	.038	1	.846	.994			
LDL	.001	.008	.006	1	.937	1.001			
Constant	2.574	1.971	1.706	1	.192	13.117			
